# Age-dependent penetrance of different germline mutations in the *BRCA1* gene

**DOI:** 10.1136/jcp.2008.062646

**Published:** 2009-03-20

**Authors:** F Al-Mulla, J M Bland, D Serratt, J Miller, C Chu, G T Taylor

**Affiliations:** 1 Department of Pathology, Molecular Pathology Unit, Faculty of Medicine, Safat, Kuwait; 2 Department of Health Sciences, University of York, York, UK; 3 Yorkshire Regional Genetics Service, St James’s Hospital, Leeds, UK; 4 St James’s Hospital Leeds, Genomic Services, Institute for Cancer Research, Cancer Research UK

## Abstract

**Aims:**

*BRCA1* gene mutations have been extensively studied in relation to breast and ovarian cancer susceptibility. Various genotype–phenotype correlation attempts have yielded important data pertaining to the consequences of *BRCA1* mutations. However, little is known about the effects of recurrent *BRCA* mutations on expressivity and the age of onset of cancer in a population. This study addresses whether different exon mutations have variable expressivity especially in relation to the age of onset of breast cancer.

**Methods:**

Using a step-wise systematic approach, culminating in the sequencing of all *BRCA1* and *BRCA2* exons with the addition of multiplex ligation-dependent probe amplification, the relationship between disease phenotypes and gene mutations in 219 individuals and their family members was examined.

**Results:**

It is shown that different *BRCA1* gene mutations have distinct effects that influence the age of onset of breast or ovarian cancer. Mutations in exon 2 of the *BRCA1* gene had significantly lower penetrance compared with mutations of exons 11, 13 and 20. The median age of affliction with breast cancer was 55 years for 185delAG in exon 2 (95% confidence interval (CI) 46.7 to 59.5), 47 years for the 4184delTCAA mutation in exon 11 (95% CI 39 to 55.4), and 41 years for exon 13 duplication (95% CI 32.9 to 49.7) of the *BRCA1* gene. Moreover, 14 novel mutations in *BRCA1* and *BRCA2* genes in the Yorkshire/Humberside population were identified.

Conclusions: The 185delAG mutation of the *BRCA1* gene is a low penetrance mutation that is age dependent especially when compared with the exon 13 duplication mutation. The data have important ramifications on screening, genetic counselling and prophylactic treatment of BRCA1 gene mutation carriers.

Breast cancer is the most prevalent malignancy in women.^1^ In the western hemisphere the lifetime risk of developing breast cancer is more than 10%. Ovarian cancer, on the other hand accounts for a lifetime risk of 1.8%.^2^ The majority of breast and ovarian cancers are sporadic or not inherited. Germline mutations in the breast and ovarian cancer susceptibility genes *BRCA1* (MIM 113705)^3^ and *BRCA2* (MIM 600185)^4^ ^5^ confer a high risk of developing breast and ovarian cancers. Women with a mutation in the *BRCA1* gene have a lifetime risk of 80–90% of developing breast cancer, and 40–65% chance of developing ovarian cancer.^6^ For *BRCA2* gene mutation carriers, the estimated cumulative risk of breast cancer has been shown to be 28% by age 50 years, and 84% by age 70 years.^7^ The risk of ovarian cancer has been shown to be 0.4% by age 50 years and 27% by age 70 years.^7^ Nevertheless, the magnitude of risk to carriers of the germline *BRCA1* and *BRCA2* remains controversial^2^ and the controversy could partly have stemmed from the use of different populations and study designs, and the lack of specific mutation stratification.^8^

The Breast Cancer Information Core Database (BIC) lists more than 1560 and 1880 mutations and polymorphisms in the *BRCA1* and *BRCA2* genes respectively. The influences of each mutation type (genotype) on expressivity, cancer type and age-dependent penetrance (phenotype) remain either controversial or unexplored. In the last decade, several reports have been published supporting the hypothesis that different mutations in the *BRCA1* or *BRCA2* genes confer different cancer-related risks. For example, Gayther *et al* (1995) reported that the risk of ovarian cancer relative to the risk of breast cancer was higher in families with mutations located 5′ to exon 13 of the *BRCA1* gene compared with families with mutations located 3′ to exon 13.^9^ However, this finding was not confirmed in later reports.^10^^–^12 Also, an ovarian cancer cluster region (OCCR) in the *BRCA2* gene was described by Gayther *et al* (1997).^13^ A later international collaborative study provided no statistically significant support for the existence of such a region,^14^ rather it was suggested that a reduced absolute risk of breast cancer for OCCR mutations contributed substantially to the effect described.^15^ All the above data pooled different but neighbouring *BRCA1* or *BRCA2* mutations and associated them with phenotypes. However, does a specific mutation in the *BRCA1* or *BRCA2* gene confer different phenotypes compared with other common mutations in the same gene? This is a question that remains unanswered especially pertaining to the recurrent *BRCA1* exon 2, 11 and 13 mutations. The answer to this question could have important implications for screening, genetic counselling and prophylactic treatment of carriers. Therefore, there is currently a need to study the genotype–phenotype correlation among common mutations in *BRCA1* and *BRCA2* genes. Our paper addresses this need.

## METHODS

### Patients

A total of 241 patients and their family members from Yorkshire and Humberside, UK, were chosen for *BRCA1* and *BRCA2* mutation testing. They belonged to 131 families classified as moderate to high risk of carrying a *BRCA1* or *BRCA2* mutation. High-risk individuals were members of families with four confirmed cases of breast and/or ovarian cancers, with breast cancer occurring under the age of 60 years or ovarian cancer at any age. Moderate-risk groups were defined as families with three cases of cancer. Patients were followed up for a mean of 81.87 months (range 0.33–343.6 months), and age at diagnosis and overall survival were recorded. Mutational and corresponding clinical data were available for 219 cases. For calculating age at diagnosis, the age and date at which cancer of the breast or ovary occurred was noted. For calculating overall survival, the date of death was recorded. In addition, for other subjects with a follow-up date, but no date of death, this was coded as censored. For one subject with no follow-up time recorded but dates of diagnosis and follow-up, the follow-up time was calculated from the dates.

All patients and family members were counselled and signed a written informed consent before testing. The procedures followed were in accordance with the ethical standards implemented at the University of Kuwait and with the Declaration of Helsinki 1975, as revised in 1983.

### Mutation testing

High-quality DNA was extracted from 5 ml EDTA-treated venous blood samples using a standard phenol–chloroform procedure. All DNA samples were screened for mutations at exon 2 (185delAG), exon 20 (5382insC) of *BRCA1*, and exon 11 (6147delT) of *BRCA2*, using amplification refractory mutation system PCR and duplication (Exon13dup6kb) of exon 13 by multiplex ligation-dependent probe amplification (P002; MRC-Holland, Amsterdam, The Netherlands) as instructed by the kit manufacturer (level 1 testing). DNA negative for these mutations was subjected to level 2 testing, which involved direct sequencing of all exon 11 in eight large PCR fragments. Negative samples were then subjected to level 3 testing that called for single-strand conformation polymorphism (SSCP) analysis and sequencing of all *BRCA1* coding exons. Therefore, all samples that were negative for levels 1, 2 and SSCP were sequenced. In all reactions, positive (DNA with known mutations) and negative controls (wild-type DNA from normal individuals) were included. Primer sequences and reaction conditions are available online.^16^

### Statistical analysis

Two outcomes were considered: age at diagnosis and time from diagnosis to death. As both of these are time to event data, Cox proportional hazards regression was used. Four groups of subjects were compared, defined by the exon where the mutation took place: exon 2, exon 11, exon 13 and all other exons. The hazard ratio for each group, exon 2, 11 and 13, compared to the other exons group were calculated, with an overall significance test of the effect of exon and confidence intervals for each hazard ratio. Patients were grouped into pedigrees, where more than one patient may belong to the same pedigree. Clustering within pedigrees was be adjusted for using robust standard errors by the method of Lin and Wei.^17^ For the time to death, we also controlled for age at diagnosis, to allow for potentially greater mortality among older women. All analyses were done using Stata 8 (Stata, College Station, Texas, USA).

## RESULTS

### *BRCA1* and *BRCA2* mutation frequencies in Yorkshire

The present study included 131 families with moderate to high risk of carrying *BRCA1* or *BRCA2* mutations (table 1). An example pedigree from a family with exon 13 duplication is shown in supplementary fig 1. A total of 241 patients and their family members were screened for pathogenic germline mutations in the *BRCA1* or *BRCA2* gene.

**Figure 1:**
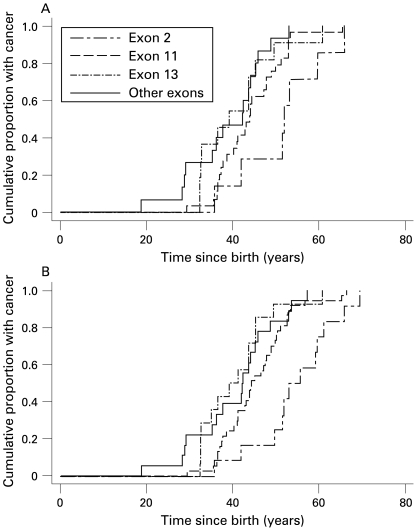
(A) Cumulative incidence for all cancer cases, by various *BRCA1* exons. (B) Cumulative incidence of breast cancer cases, by various *BRCA1* exons.

**Table 1:** Breakdown of families with the most frequent *BRCA1* mutations

Family	Mutation	Gene/exon	No of individuals	No of mutation carriers	No affected
761	185delAG	*BRCA1/2*	4	1	0
2802	185delAG	*BRCA1/2*	26	11	3
5358	185delAG	*BRCA1/2*	2	1	1
9603	185delAG	*BRCA1/2*	1	1	0
9877	185delAG	*BRCA1/2*	4	4	1
12654	185delAG	*BRCA1/2*	1	1	1
13376	185delAG	*BRCA1/2*	2	1	1
17240	185delAG	*BRCA1/2*	2	2	2
17924	185delAG	*BRCA1/2*	2	1	1
21347	185delAG	*BRCA1/2*	2	2	2
27388	185delAG	*BRCA1/2*	4	2	1
30231	185delAG	*BRCA1/2*	2	1	1
2592	4184delTCAA	*BRCA1/11*	3	2	1
7836	4184delTCAA	*BRCA1/11*	4	3	1
9117	4184delTCAA	*BRCA1/11*	4	2	1
9430	4184delTCAA	*BRCA1/11*	3	2	2
10079	4184delTCAA	*BRCA1/11*	3	2	1
10502	4184delTCAA	*BRCA1/11*	2	1	1
11096	4184delTCAA	*BRCA1/11*	3	1	1
12779	4184delTCAA	*BRCA1/11*	3	1	1
14028	4184delTCAA	*BRCA1/11*	2	2	2
14562	4184delTCAA	*BRCA1/11*	3	2	1
16324	4184delTCAA	*BRCA1/11*	1	1	1
29694	4184delTCAA	*BRCA1/11*	7	4	3
32778	4184delTCAA	*BRCA1/11*	1	1	1
33164	4184delTCAA	*BRCA1/11*	1	1	1
39265	4184delTCAA	*BRCA1/11*	3	3	3
40426	4184delTCAA	*BRCA1/11*	1	1	0
3140	Exon13 Dup	*BRCA1/13*	3	2	1
3429	Exon13 Dup	*BRCA1/13*	2	2	1
9592	Exon13 Dup	*BRCA1/13*	3	1	1
12157	Exon13 Dup	*BRCA1/13*	1	1	Unknown
12468	Exon13 Dup	*BRCA1/13*	2	2	1
20332	Exon13 Dup	*BRCA1/13*	2	1	1
20597	Exon13 Dup	*BRCA1/13*	1	1	1
21231	Exon13 Dup	*BRCA1/13*	2	1	1
25940	Exon13 Dup	*BRCA1/13*	2	2	1
27810	Exon13 Dup	*BRCA1/13*	2	1	1
32563	Exon13 Dup	*BRCA1/13*	2	2	2
36387	Exon13 Dup	*BRCA1/13*	1	1	1
38199	Exon13 Dup	*BRCA1/13*	2	1	1

One hundred and thirty-six (56%) individuals carried a mutated *BRCA1* gene, 25 (10%) had a mutated *BRCA2* gene, and 80 (33%) of the screened individuals carried wild-type genes. Tables 2 and 3 give the frequency and type of mutations in various exons of *BRCA1* and *BRCA2* genes respectively. The vast majority of mutations caused protein truncation. Moreover, we identified 14 novel mutations not previously reported in the BIC database.^18^

**Table 2:** Frequencies and types of *BRCA1* familial mutations

Exon no	Familial mutation	No of individuals (%) with*BRCA1* mutations (n = 136)
11	917_918delTT	1 (1.7)
	887insAG*	1 (1.7)
	887insA*	1 (1.7)
	4184delTCAA	29 (49.2)‡
	3896delT	5 (8.5)
	3889delAG	3 (5.1)
	3875delGTCT	2 (3.4)
	3519G>T (E1134X)	1 (1.7)
	3450_3453delCAAG	1 (1.7)
	2871A>C*	1 (1.7)
	2800delAA	1 (1.7)
	2682C>T (Q855X)	1 (1.7)
	2594delC	2 (3.4)
	2559insA*	1 (1.7)
	2196G>A (D693N)	1 (1.7)
	2080delA	1 (1.7)
	2011insT	1 (1.7)
	1927C>G*	1 (1.7)
	1623del5bp	2 (3.4)
	1294del40bp	2 (3.4)
	Total	58
13	Ex13ins6kb	4 (20)
	ex13 dup	14 (70)§
	4446C>T (R1443X)	2 (10)
	Total	20
15	4693delAA	1 (50)
	4654G>T (S1512I)	1 (50)
	Total	2
16	5075G>A (M1652I)	1 (100)
	Total	1
1A–2	Exons 1A–2 deletion	2 (100)
	Total	2
2	187–188delAG*	1 (3.3)
	185insA†	1 (3.3)
	185delAG	28 (93.3)¶
	Total	30
20	ivs20+60 ins12bp	1 (8.3)
	Exon 20 deletion	7 (58.3)
	5382insC	4 (33.3)
	Total	12
22	5452–2A>C*	1 (100)
	Total	1
24	5622C>T (R1835X)	5 (100)
	Total	5
3	Exon 3 deletion	3 (100)
	Total	3
5	300T>C (C61R)	1 (100)
	Total	1
7	421–2delA	1 (100)
	Total	1

*Novel mutations not reported in the Breast Cancer Information Core database.

†Reported in Pakistan.^36^

‡Number of families = 13.

§Number of families = 12.

¶Number of families = 11.

**Table 3:** Frequencies and types of *BRCA2* familial mutations

Exon no	Familial mutation	No of individuals (%) withBRCA2* mutations (n = 25)
11	6714_6717delACAA	2 (11.8)
	6503delTT	1 (5.9)
	6174delT	1 (5.9)
	6137C>A (S1970X)	2 (11.8)
	5950delCT	2 (11.8)
	5368delATTT*	1 (5.9)
	5056insG*	3 (17.6)
	4859insA	2 (11.8)
	4329insA*	1 (5.9)
	4329delA*	1 (5.9)
	3386T>G (L1053X)	1 (5.9)
	3036delACAA	1 (5.9)
	Total	18
2	295G>T	4 (100)
	Total	4
23	9138insA*	1
	Total	1
8	860–1G>A*	2 (100)
	Total	2

*Novel mutations not reported in Breast Cancer Information Core database.

For the *BRCA1* gene, mutations in exon 11 were the most frequent (n = 58, or 42.3%) with 4184delTCAA accounting for the most common mutation within exon 11 (n = 29, or 50%). Exon 2 mutations were the second most frequent (n = 30, or 22%) with 185delAG being the most common mutation within exon 2 (n = 28, or 93.3%) followed by mutation of exon 13 (n = 20, or 14.6%) and exon 20 (n = 12, or 8.7%). For *BRCA2* gene, mutations in exon 11 were the most frequent (n = 18, or 75%).

### Association of *BRCA1* and *BRCA2* gene mutations with cancer occurrence and site

Matched mutations and clinical data were available for 219 individuals. The cohort included 40 (18%) males and 179 (82%) females. As expected, there was a significant association between the presence of *BRCA1* or *BRCA2* mutations and the occurrence of cancer; p<0.0001 (table 4). Of the affected cases, 63 had invasive breast cancer, 12 were diagnosed with ovarian cancer, 15 had both breast and ovarian cancers, one man developed colon and prostate cancers, one woman presented with ovarian and vaginal cancers, and another woman presented with breast and pancreatic adenocarcinomas. Table 5 lists cancer sites in relation to *BRCA1* and *BRCA2* mutations. Mutations in the *BRCA1* gene accounted for the majority of breast and ovarian cancers in these families. In addition, *BRCA1* mutations accounted for the majority of patients with both breast and ovarian cancers. *BRCA2* mutations were more common in breast than ovarian cancer (the ratio of breast to ovarian cancers was 9:1). This is consistent with data from the Breast Cancer Linkage Consortium.^7^ Although the numbers were modest, mutations in the *BRCA2* gene were significantly associated with the development of cancers outside the breast–ovarian axis (table 5).

**Table 4:** Clinical characteristics of patients in relation to *BRCA1* and *BRCA2* mutations

Clinical data	Number of patients or age	Mutations in*BRCA1* and*BRCA2* genes	Wild-type*BRCA1* and*BRCA2* genes	p Value*
Phenotype				
No with cancer	102	98	4	0.0001
No cancer free	117	44	73	
Mean age (years)	47.7			
Mean age of patients with cancer	46	45.8	49.2	0.006†
Mean age of cancer-free individuals	49.2	46.7	50.8	

*Calculated using two-sided Fisher’s exact test.

†Student t test was used to compare means.

**Table 5:** Cancer sites in relation to *BRCA1* and *BRCA2* mutations

Cancer site*	Mutation in*BRCA1*	Mutation in*BRCA2*	Total	p Value†
Breast	54	9	63	0.001
Ovary	12	0	12	
Breast and ovary	13	2	15	
Breast or ovary and other sites	0	2	2	
Other sites	0	1	1	
Total	79	14	93	

*Site was unknown for five of 98 patients with cancer (three with exon 11, and two other exon mutations in the *BRCA1* gene).

†Calculated using two-sided Fisher’s exact test.

There was evidence that mutations in the 3′ end of the *BRCA1* gene (3′ to exon 13) were less likely to be associated with ovarian cancer. For example, 90% of ovarian cancers were associated with mutations in the 5′ end of the *BRCA1* gene, while only 10% were linked to mutations in the 3′ end. However, this trend was not statistically significant.

### *BRCA1* exon 2 mutation penetrance

We noted that carriers of the exon 2 *BRCA1* gene mutation had significantly lower cancer incidence compared with carriers of mutations of exons 11, 13 and 20. In this regard, table 6 shows that 15 (56%) of patients who carried the *BRCA1* 185delAG mutation were cancer free (p = 0.004). This suggested to us that either the 185delAG mutation is of low penetrance or, more likely, carriers of this mutation would develop cancer at an older age compared with other *BRCA1* gene mutations. This observation led us to explore age-dependant expressivity of various *BRCA1* exon mutations.

**Table 6:** Relationship between various mutated *BRCA1* exons and affliction with cancer

Mutated exon no.	Affected with cancer, n = 84 (%)	Cancer free, n = 37 (%)	Total, n = 121
11	38 (71.7)	15 (28.3)	53
13	13 (72.2)	5 (27.8)	18
2	12 (44.4)	15 (55.6)*	27
Other exons	21 (91)	2 (9)	23

*p = 0.004 using two-sided Fisher’s exact test.

### *BRCA1* mutations and age-related expressivity

Of the 136 cases carrying a mutated *BRCA1* gene, 79 women who developed breast and/or ovarian cancers were included in the analysis (table 7). Analysis of age-related expressivity pertaining to various *BRCA2* gene mutations was not performed because of the small number of cases included in each mutation category. For *BRCA1* gene, groups of patients were compared, defined by the exon where the mutations were located: exon 2, exon 11, exon 13, and all other exons. Patients were grouped into pedigrees, where more than one patient may belong to the same pedigree. Cox regression with clustering on pedigree showed that the exon was a highly significant predictor (χ^2^ 19.75 with 3 degrees of freedom, p = 0.0002). Hence, there was a significant relationship between a specific exon mutation and age at diagnosis, mainly due to exon 2 having a much lower hazard or older age at diagnosis than the other groups (table 7). Clustering within pedigrees may have an adverse effect on p values and standard errors. The cumulative incidence for all cancer cases, or limited to breast cancers, and stratified by various *BRCA1* exon mutations is shown in fig 1.

**Table 7:** Cox regression analysis between specific *BRCA1* exon mutation and age at diagnosis

Mutation	Hazard ratio	Standard error	p Value (95% CI)
Exon 11	0.69	0.21	0.2 (0.38 to 1.25)
Exon 2	0.28	0.09	<0.001 (0.15 to 0.54)
Exon 13	1.16	0.51	0.7 (0.49 to 2.75)

Hazard ratio of <1 indicates lower hazard than the other groups and hence older ages at diagnosis.

We then asked if the relationship between various *BRCA1* exon mutations and age at diagnosis is different for different cancer sites. For this, we carried out Cox regression analysis with exon and tumour site as factors. The estimates of the hazard ratios were very little changed by including both exon and tumour site (χ^2^ statistic 23.09 with 5 degrees of freedom, p = 0.0003) (table 8). Also, limiting the analysis to woman with breast cancer only, the hazard ratios changed very little (table 9). This is not surprising, as most of the women belonged to this group. Hence there is strong evidence that various *BRCA1* exon mutations have an effect on age-dependent expressivity that was not explained by cancer site.

**Table 8:** Cox regression analysis including specific *BRCA1* exon mutation and cancer sites as covariates

Mutation	Hazard ratio	p Value (95% CI)
Exon 11	0.64	0.1 (0.37 to 1.11)
Exon 2	0.29	<0.001 (0.15 to 0.53)
Exon 13	0.98	0.97 (0.40 to 2.42)
Ovary	0.46	0.009 (0.26 to 0.82)
Other exons	0.76	0.3 (0.47 to 1.24)

**Table 9:** Cox regression analysis between specific *BRCA1* exon mutation and age at diagnosis limited to breast cancer only

Mutation	Hazard ratio	p Value (95% CI)
Exon 11	0.61	0.1 (0.32 to 1.13)
Exon 2	0.26	0.002 (0.11 to 0.62)
Exon 13	0.80	0.6 (0.32 to 2.03)

### Specific *BRCA1* mutations and age-related expressivity

**Figure 2:**
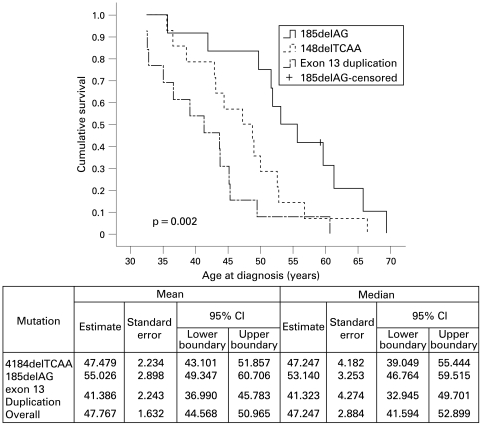
Kaplan–Meier plots of cumulative survival and age at diagnosis with breast cancer in relation to specific *BRCA1* exon mutations. The p value indicates log-rank test.

Exon 11 is the largest exon in the *BRCA1* gene and harbours a number of different mutations in different patients; these mutations were grouped in this study under the term “exon 11 mutations”. To minimise any confounding error emanating from such grouping, we limited the analysis to three specific and most common mutations in exons 2, 11 and 13, namely, 185delAG, 4184delTCAA and exon 13 duplication, respectively (table 1). Figure 2 shows that median age of affliction with breast cancer was 55 years for 185delAG in exon 2 (hazard ratio (HR) 0.23, 95% CI 0.095 to 0.55, p = 0.001) and 47 years for the 4184delTCAA mutation in exon 11 (HR 0.49, 95% CI 0.225 to 1.08, p = 0.076) compared with 41 years for exon 13 duplication of the *BRCA1* gene. It is worthy of note that the inclusion of a large pedigree (2802) carrying the 185delAG mutation could have confounding influence on the major conclusion emanating from our study. However, exclusion of this pedigree from the analysis did not have any impact on our data stating that exon 2 mutation 185delAG has lower penetrance that is age dependent (HR 0.22, 95% CI 0.09 to 0.56, p = 0.002).

### *BRCA1* mutations and survival analysis

**Figure 3:**
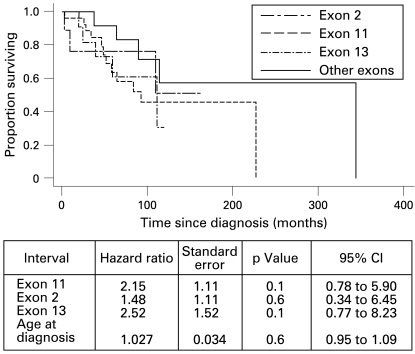
Kaplan–Meier plot of overall survival of patients in relation to *BRCA1* exon mutations (the table depicts the hazard ratios).

Overall survival data were available from 61 patients. The Cox regression with clustering on pedigree, including *BRCA1* exon mutation and age at diagnosis, gave no overall significant prediction: χ^2^ = 3.72 with 4 degrees of freedom, p = 0.4. Thus there was no evidence that any specific *BRCA1* exon mutation can predict overall survival from date of diagnosis. However, as expected, risk of death increased slightly with age at diagnosis, though this was not significant. The raised HRs suggest that this analysis would be worth repeating with a much larger sample (fig 3).

## DISCUSSION

The aim of this study was to elaborate on the expressivity of various *BRCA1* gene mutations in relation to penetrance and age of cancer diagnosis. To this end, a total of 241 patients and their family members (of whom 219 had relevant clinical data) were screened for pathogenic germline mutations in *BRCA1* or *BRCA2* genes and the results were associated with clinical parameters. We opted for a family-based study to maximise the number of individuals with mutated *BRCA1* or *BRCA2* genes. Although this approach suffers from bias compared with a population-based study, it was necessary as the latter approach requires a very large cohort to attain a similar number of individuals with mutated *BRCA1* or *BRCA2* genes. Therefore, >4000 individuals in a population are required to be tested to yield 141 individuals with *BRCA1* or *BRCA2* mutations.^19^ The extent of bias is difficult to infer. For example, it is generally accepted that family-based studies usually overestimate the lifetime risks of cancer because the presence of additional genetic factors could elevate risks for the relatives. Nevertheless, the use of appropriate statistical methods could alleviate some aspects of this bias.^20^ ^21^

In this study we have exhaustively searched for mutations in the *BRCA1* and *BRCA2* genes using a level approach that involved starting from screening for known founder mutations up to sequencing all exons in addition to various exon rearrangements. This methodology would increase the sensitivity of detecting *BRCA1* and *BRCA2* gene mutations, and this was reflected in our high pickup mutation rate accounting for 67% of individuals recruited in this study. A total of 56% of individuals harboured a *BRCA*1 mutated gene; this figure is similar to the one previously estimated for the UK population.^9^ Similarly, the proportion of *BRCA2* mutation-bearing individuals in this study is within the range previously published for the UK.^22^ It is well accepted that mutation frequencies of the *BRCA1* and *BRCA2* genes in high-risk families vary widely among different populations, being highest in Russia for *BRCA1*^23^ and in Iceland for *BRCA2*.^24^ This could be due to ascertainment or methodological bias, or more likely it is a reflection of human migration and establishment of founders. In our UK East Midlands series, three mutations in the *BRCA1* gene were recurrent, namely 4184delTCAA, accounting for the most common mutation within exon 11 (found in 16 families), 185delAG, being the most frequent mutation within exon 2 (found in 11 families), and duplication of exon 13 (found in 12 families). The BIC database lists 185delAG, 4184delTCAA and exon 13 duplication among the 20 most frequent recurrent mutations^18^; these are consistent with our data. Also, our data are in line with the fact that *BRCA1* exon 13 duplication has been reported as a founder mutation in Britain, and English-speaking counties with historical links to Britain. In that regard, 50% of the exon 13 duplication mutations were demonstrated in Britain, mainly Yorkshire, suggesting a northern British origin for this mutation (the *BRCA1* exon 13 screening group).^25^ Interestingly, none of the *BRCA2* mutations were repeated in more than two families, probably indicating the presence of many *BRCA2* founders (or de novo mutations). Although we did not haplotype families carrying the 185delAG mutation, previous haplotypes from Yorkshire indicated independent origins of the mutation from the Jewish founders.^26^

Our data are consistent with the fact that mutations in *BRCA1* and *BRCA2* are associated with increased risk of cancer.^3^ ^27^ Both *BRCA1* and *BRCA2* mutations were significantly associated with development of breast cancer. On the other hand, *BRCA2*, but not the *BRCA1* gene, was significantly linked to the development of cancer outside the breast–ovarian axis. This is in agreement with data showing that mutations in the *BRCA1* gene are rarely linked to cancer at sites other than breast and ovary, while mutations in the *BRCA2* gene are.^28^ ^29^

Regarding the controversial issue linking mutations of the 3′ end of the *BRCA1* to lower incidence of ovarian cancer, our data show that ovarian cancer is indeed lower when mutations inflict the 3′ end of the *BRCA1* gene. However, the trend was insignificant and a larger study is needed to resolve this issue.^9^

Perhaps the most intriguing finding of this study was the notion that different mutations in the *BRCA1* gene have significantly different effect on age-dependent expressivity. To the best of our knowledge this is the first time that different age-dependent expressivity, independent of cancer site, has been reported for three of the most common *BRCA1* mutations, namely 185delAG, 4184delTCAA and exon 13 duplication. For the exon 13 duplication mutation, we reported a mean age of cancer diagnosis at 41.4 years (95% CI 36.99 to 45.78) from 12 independent families. Data on the age-dependent penetrance/expressivity for exon 13 duplication are scarce. However, Unger *et al* reported a Belgian woman with this mutation who developed cancer at age 34 years.^30^ Moreover, Yap *et al* reported a woman diagnosed with bilateral breast cancer at ages 32 and 41 years and her sister, carrying the same exon 13 duplication mutation, was diagnosed with ovarian cancer at age 43 years.^31^ For the 185delAG *BRCA1* mutation, although the frequency of the mutation was, as expected, among the highest, we report significantly lower cancer rates in carriers of this mutation. This has been observed before in Iraqi/Iranian women, who have a significantly lower penetrance compared with Ashkenazi Jewish woman carrying the same mutation.^32^ Therefore, it remains to be elucidated whether this difference in penetrance could be due to other genetic variation such as different haplotypes, environmental or cultural influences. More importantly, we report here that the mean age of cancer diagnosis from 12 separate families carrying the 185delAG mutation was 55 years (95% CI 49.3 to 60.7), which is a significantly older age of cancer diagnosis compared with exon 13 duplication mutation. In a recent report, using a meta-analysis of pedigree data from 498 *BRCA1* and *BRCA2* mutation carriers identified through population-based studies of women with breast and ovarian cancer, the authors have shown, albeit not significantly, that cancer risk by age 50 years is lower for 185delAG than 5382insC *BRCA1* mutation carriers.^19^ ^33^ Our data are consistent with this. The question of why the 185delAG mutation has a lower cancer-related expressivity that is age dependent compared to other *BRCA1* mutations remains to be elucidated. Recently, however, Perrin-Vidoz *et al*, reported that transcripts bearing the 185delAG mutation are not subjected to degradation by nonsense-mediated mRNA decay.^34^ This important observation leads us to hypothesise that even in the presence of the 185delAG mutation there could still be some truncated, but functional, BRCA1 protein delaying the onset of cancer. In support of this, Buisson *et al* have demonstrated translational re-initiation in the *BRCA1* minigenes at position 128 in the presence of a premature termination codon at position 36 or 39.^35^ However, using western blotting and immunoprecipitation, we and Buisson *et al* were unable to detect N-terminally or C-terminally truncated BRCA1 proteins from lymphocytes of carriers, probably indicating an unstable nature of the truncated BRCA1 protein.^35^ Nevertheless, the truncated BRCA1 protein could still be functional in a microenvironment or at concentrations beyond the detection limit of current technologies. This hypothesis remains to be proven. Obviously, the role of other genetic background or haplotypes and environmental influences on age-dependent expressivity cannot be underestimated. For example, we do not know whether carriers of exon 13 duplication in the *BRCA1* gene have other genetic aberrations that could influence expressivity. Regardless of the mechanism, our findings have important ramifications regarding the design of future studies for the estimation of cancer risks and more specifically on the counselling of patients with *BRCA1* mutations, in that the mutation type should be included in risk calculations. Moreover, the time of screening and prophylactic treatments of carriers should be adjusted to take into account the different age-dependent expressivity of various *BRCA1* gene mutations.

**Take-home messages**

- The 185delAG mutation of the *BRCA1* gene is a low penetrance mutation that is age dependent.
- Carriers of the exon 13 duplication mutation are at risk of developing cancer at younger age.
- We report 14 novel mutations in the *BRCA1* and *BRCA2* genes in the Yorkshire/Humberside population.
- Clinical geneticists should take the mutation site and type into consideration in risk calculations.
